# Genomic epidemiology of *Shigella* in the United Kingdom shows transmission of pathogen sublineages and determinants of antimicrobial resistance

**DOI:** 10.1038/s41598-018-25764-3

**Published:** 2018-05-09

**Authors:** Kate S. Baker, Timothy J. Dallman, Nigel Field, Tristan Childs, Holly Mitchell, Martin Day, François-Xavier Weill, Sophie Lefèvre, Mathieu Tourdjman, Gwenda Hughes, Claire Jenkins, Nicholas Thomson

**Affiliations:** 1 Institute for Integrative Biology, University of Liverpool, Liverpool, L69 7ZB United Kingdom; 2 Gastrointestinal Bacterial Reference Unit, National Infection Service, Public Health England, London, NW9 5HT United Kingdom; 3 Centre for Molecular Epidemiology and Translational Research, Institute for Global Health, UCL, London, United Kingdom; 4 Centre for Infectious Disease Surveillance and Control, National Infection Service, Public Health England, London, NW9 5HT United Kingdom; 5 Institut Pasteur, Unité des Bactéries Pathogènes Entériques, Paris, 75015 France; 6 Santé Publique France, the French Public Health Agency, Saint-Maurice, France; 7 Department of HIV and STIs, National Infection Service, Public Health England, London, NW9 5HT United Kingdom; 8 Wellcome Trust Sanger Institute, Hinxton, CB10 1SA United Kingdom; 9 London School of Hygiene and Tropical Medicine, London, WC1E 7HT United Kingdom

## Abstract

Shigella are globally important diarrhoeal pathogens that are endemic in low-to-middle income nations and also occur in high income nations, typically in travellers or community-based risk-groups. Shigella phylogenetics reveals population structures that are more reliable than those built with traditional typing methods, and has identified sublineages associated with specific geographical regions or patient groups. Genomic analyses reveal temporal increases in *Shigella* antimicrobial resistance (AMR) gene content, which is frequently encoded on mobile genetic elements. Here, we whole genome sequenced representative subsamples of *S*. *flexneri* 2a and *S*. *sonnei* (n = 366) from the United Kingdom from 2008 to 2014, and analysed these alongside publicly available data to make qualitative insights on the genomic epidemiology of shigellosis and its AMR within the broader global context. Combined phylogenetic, epidemiological and genomic anlayses revealed the presence of domestically-circulating sublineages in patient risk-groups and the importation of travel-related sublineages from both Africa and Asia, including ciprofloxacin-resistant sublineages of both species from Asia. Genomic analyses revealed common AMR determinants among travel-related and domestically-acquired isolates, and the evolution of mutations associated with reduced quinolone susceptibility in domestically-circulating sublineages. Collectively, this study provides unprecedented insights on the contribution and mobility of endemic and travel-imported sublineages and AMR determinants responsible for disease in a high-income nation.

## Introduction

*Shigella* are the most common bacterial cause of moderate to severe diarrhoea in children under 5 years old in Asia and Africa^1^. In high income nations, shigellosis is often related to travel but can also circulate as a sexually transmissible illness of men who have sex with men (MSM)^2,3^. Among the four species comprising the genus *Shigella, S*. *sonnei* and *S*. *flexneri* cause the greatest disease burden. Among the diverse (>50) serotypes of *S*. *flexneri*, the 2a serotype is historically the most prevalent worldwide and in the United Kingdom (UK)^4,5^. However, increased resolution of *Shigella* species population structures has been provided by Whole Genome Sequence Analysis (WGSA). *S*. *sonnei* is split into five WGSA subtypes called ‘lineages’, with the original four lineages^6^ being joined recently by a fifth reported in Latin America and Africa^7^. *S*. *flexneri* has been similarly divided into seven WGSA subtypes called ‘phylogroups’ (PGs)^8^. In addition to these species-defining studies, prevailing and emerging lower-order WGSA subtypes (usually called ‘sublineages’) of *S*. *sonnei* and *S*. *flexneri* have also been described in countries in Asia, the Middle East and Latin America^7,9–12^. WGSA has also been used to demonstrate the international movement of *Shigella* sublineages where WGSA subtypes prevailing in one geographical region are found in another, isolated from patients with an epidemiological link (e.g. travel)^13,14^. For example, a specific sublineage of *S*. *sonnei* that dominates in Israel circulates globally among Orthodox Jewish communities^10^.

On top of this global population structure of shigellae WGSA subtypes, there is a horizontal and vertical transmission network of genes, including those encoding antimicrobial resistance (AMR). When WGSA subtyping is combined with epidemiological metadata and genome analyses (genomic epidemiology), the importance of AMR in shaping epidemic emergences and broader population structure in shigellae has been revealed. Studies over long time scales have shown the accumulation of AMR in *Shigella* over time^8,15,16^, and both local and global epidemic emergences of WGSA subtypes have been found to be associated with resistance to therapeutically-important antimicrobial classes^6,9,12,13^. AMR determinants in *Shigella* include Mobile Genetic Elements (MGEs), such as small and large resistance plasmids, and chromosomally-integrated islands, some of which have been found in multiple *Shigella* species. For example, the *Shigella* Resistance Locus Multiple Drug Resistance Element (SRL-MDRE) encoding resistance to ampicillin, chloramphenicol and tetracycline has been found in widely geographically-distributed *S*. *dysenteriae*, *S*. *flexneri* and *S*. *sonnei*^7,8,15,17^. In addition to these horizontally-acquired AMR determinants, chromosomal mutations in the Quinolone Resistance Determining Region (QRDR) (that confer resistance to ciprofloxacin, the recommended treatment for shigellosis)^18^ have also been reported in shigellae isolated from Asia, Latin America, and high-income nations^7,11,14,19^. Ciprofloxacin-resistant *Shigella* were recently recognised by the World Health Organisation as one of the top dozen agents for which new antimicrobials are urgently needed. Common AMR determinants being found in geographically-separated and genetically-distinct *Shigella* subtypes point to the independent mobility of AMR determinants as well as convergent evolution.

With an increased resolution and volume of information on the population structure of *Shigella* and its AMR determinants available, these data can be used to enhance surveillance in public health laboratories that are using whole genome sequencing, such as the Gastrointestinal Bacteria Reference Unit (GBRU) at Public Health England (PHE) in the UK^20^. In the UK, shigellosis has historically been associated with travel and only low-levels of domestic acquisition (i.e. illness in patients without a recent history of travel to a shigellosis-endemic area). Recently however, epidemics of domestically-acquired shigellosis (*S*. *sonnei, S*. *flexneri* 2a and *S*. *flexneri* 3a) have been linked with transmission among adult males^21^, and more specifically among men who have sex with men (MSM)^5,22^. As part of a recent study investigating AMR emergence among these parallel epidemics in MSM^23^, we whole genome sequenced representative subsamples of *S*. *sonnei* (n = 187) and *S*. *flexneri* 2a (n = 179) from the UK sampled between 2008 and 2014. Here, in this allied study, we integrate this new cross sectional data from the UK with existing reference datasets of *Shigella* WGSA subtypes and AMR determinants from around the world to identify those responsible for shigellosis in the UK. By providing the context necessary for interpreting national surveillance patterns, we reveal important qualitative insights on the importation and domestic transmission of *Shigella* in the UK, as well as on the prevalence and mobility of AMR among these globally-important pathogens.

## Methods

### Nationally representative isolates

**Figure 1:**
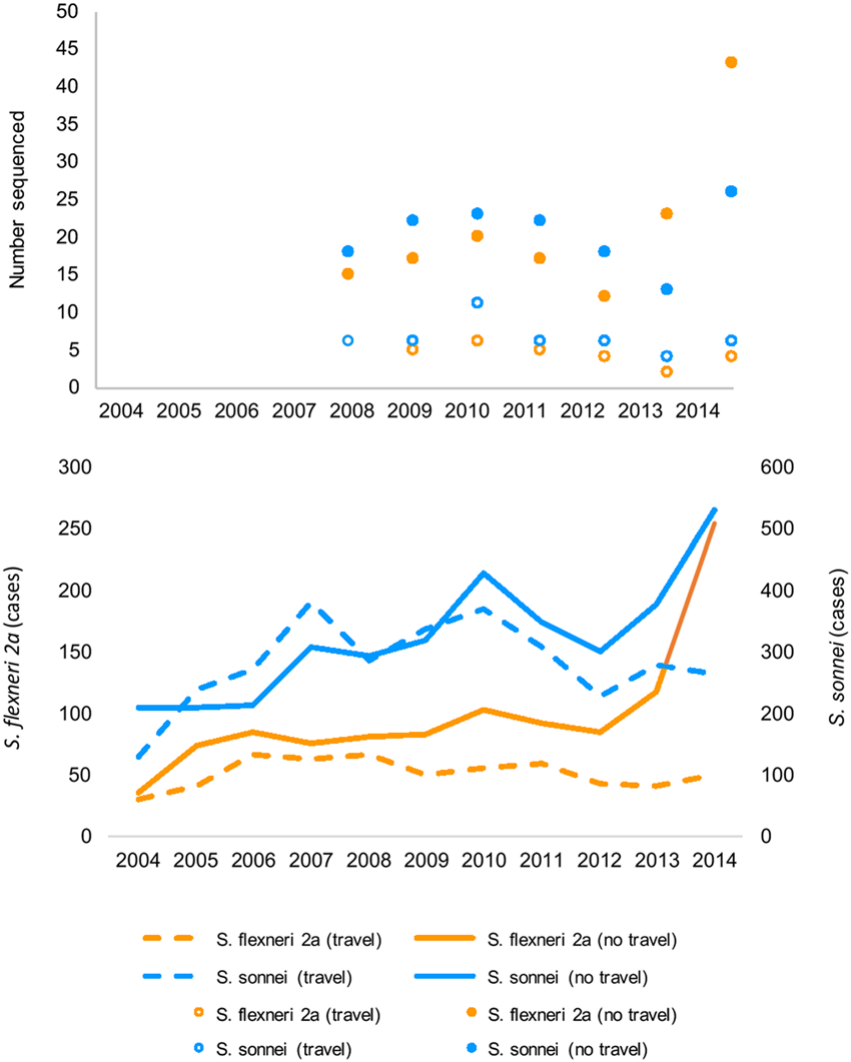
Representative sampling of isolates for whole genome sequencing. The figure shows a graph of the number of isolates sequenced per year by travel and species (upper graph), overlaid with the number of domestically-acquired (non-travel) and travel-associated cases of shigellosis (for all ages). Non-travel isolates were overrepresented (see Methods).

To investigate an epidemic phase of *Shigella* transmission associated with MSM^21,23^, isolates of *S*. *sonnei* and *S*. *flexneri* 2a submitted to the GBRU at PHE were representatively subsampled, with a random sample of isolates from each year proportional to the total number of annual cases (for each species) being taken from across 2008–2014 (Fig. 1). In doing so, we aimed to balance an economically-practical number of isolates for WGSA, with the need to have sufficient statistical power to detect emerging sublineages of domestic transmission (this calculation was based on data in^13,21^). For *S*. *flexneri* 2a, there were no selection criteria based on age, whereas for *S*. *sonnei* (owing to the larger number of cases that occur annually) selection was restricted to patients aged 16–60 years old. To focus on the epidemic domestic transmission, we sequenced a greater proportion of isolates from patients who did not report recent high-risk travel for both species (Fig. 1). The final proportions were as follows: for *S*. *sonnei* 3% (45 of 1539 cases) of travel-associated cases and 8% (142 of 1751 cases) of non-travel associated cases from patients aged 16–60 years old, and for *S*. *flexneri* 2a 9% (32 of 368 cases) of travel-associated cases and 18% (147 of 820 cases) of non-travel associated cases from patients of all ages. This resulted in a total of 187 *S*. *sonnei* and 179 *S*. *flexneri* 2a isolates (Fig. 1).

### Publically available data

Various *S*. *sonnei* reference isolates (n = 40) from other WGSA studies were included to provide interpretative context. Representative contextual isolates and the associated studies were as follows: the original four global lineages of *S*. *sonnei*, including those classified into the multidrug resistant and globally-disseminated ‘Global III’ sublineage^6^; the newly identified global Lineage V and a regionally-epidemic Latin America sublineage, Latin America IIIa^7^; a sublineage associated with transmission in Israel and Orthodox Jewish communities elsewhere^10^; and a ciprofloxacin-resistant sublineage circulating in, and disseminating from, Asia, the ‘Central Asia III’ lineage^14^ (Supplementary Data). For *S*. *flexneri* 2a, isolates sequenced in a population-structure-defining study^8^ were included in analyses to anchor the positions of the *S*. *flexneri* 2a representative subsample, and investigate geographical associations. Specifically, this included representatives of all PGs (n = 9) and all PG3 *S*. *flexneri* 2a isolates (n = 69) (Supplementary Data).

### Bioinformatic analyses

DNA was extracted and isolates whole genome sequenced as previously described^13^. Whole genome sequence phylogenetic trees were constructed through mapping of sequence data (using smalt, http://www.sanger.ac.uk/science/tools/smalt-0) to reference genomes (*S*. *flexneri* 2a 2457 T, *S*. *sonnei* 53 G), followed by removal of mobile (plasmids, insertion sequences, other described mobile islands^6,24^) and repetitive elements (through removal of known repeat regions and repeat mapping discard), and sites of recombination (using gubbins^25^), followed by extraction of variable sites (using snp_sites^26^). This resulted in multiple sequence alignments of 19348, 11141, and 9790 variant sites in length for *S*. *flexneri* (all PGs), *S*. *flexneri* (PG3 only), and *S*. *sonnei* respectively. Multiple sequence alignments were used to infer maximum likelihood phylogenetic trees using RAxML with 100 bootstrap replicates^27^. Acquired AMR genes were identified using ResFinder^28^ on draft genome assemblies (generated by^29^) and QRDR mutations were detected as previously described^7^. All sequence data are publically available at the European Nucleotide Archive under project number PRJEB12097 (individual accessions in Supplementary Data).

### Ethical statement

No individual patient consent was required or sought as PHE has authority to handle patient data for public health monitoring and infection control under section 251 of the UK National Health Service Act of 2006 (previously section 60 of the Health and Social Care Act of 2001). The project was reviewed and approved by the PHE Research Support and Governance Office and Caldicott Panel deemed to comply with public health surveillance standards.

### Data availability statement

All sequencing and genome assembly data for isolates in this study are available under project number PRJEB12097 in the European Nucleotide Archive. Further detail linking isolates and sample accession numbers within the project are available in Supplementary Data.

## Results

### Mobility of *S. sonnei* and *S. flexneri* 2a WGSA subtypes

**Figure 2:**
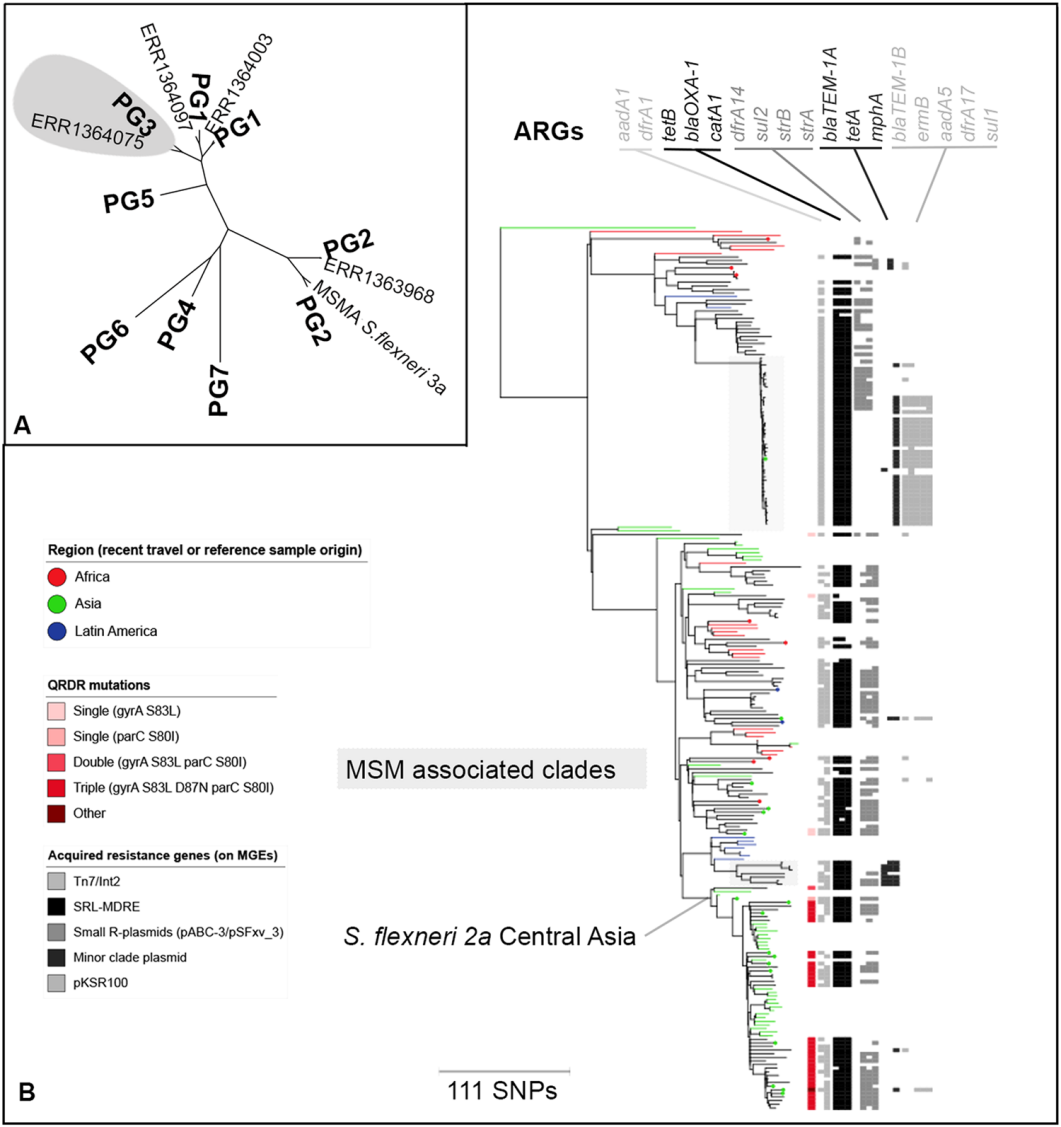
*Shigella flexneri* 2a UK cross section in context. (**A**) The smaller tree shows the broad context of phylogroups (PG) 1–7 of *S*. *flexneri* and intercontinental MSM-associated (MSMA) *S*. *flexneri* 3a, with the relationships of three non-PG3 isolates from this study. PG3 is overlaid by a grey polygon in the smaller tree and is shown in full in the larger tree. (**B**) The larger tree is a mid-point rooted maximum likelihood tree and shows the detailed evolutionary relationships of UK cross section isolates (black branches) with reference isolates (coloured branches). Reference isolate branches are coloured by region of sample origin, and region of recent travel is overlaid for UK samples as coloured circles on tree tips (both coloured according to the inlaid region key). Two MSM-associated sublineages are highlighted by grey boxes, and the *S*. *flexneri* 2a Central Asia lineage is indicated at the defining internal node. AMR features are shown in adjacent tracks. The leftmost track shows the presence of QRDR mutations and one ‘Other’, which designates a quadruple mutant also carrying the *qnrS1* gene. Subsequent gray scale tracks indicate the presence of antimicrobial resistance genes (ARGs, labelled above) associated with known *Shigella* MGEs. Specifically (from left to right), the Tn7/Int2, SRL-MDRE, Small R-plasmids, Minor clade plasmid, pKSR100 (Table 1).

**Figure 3:**
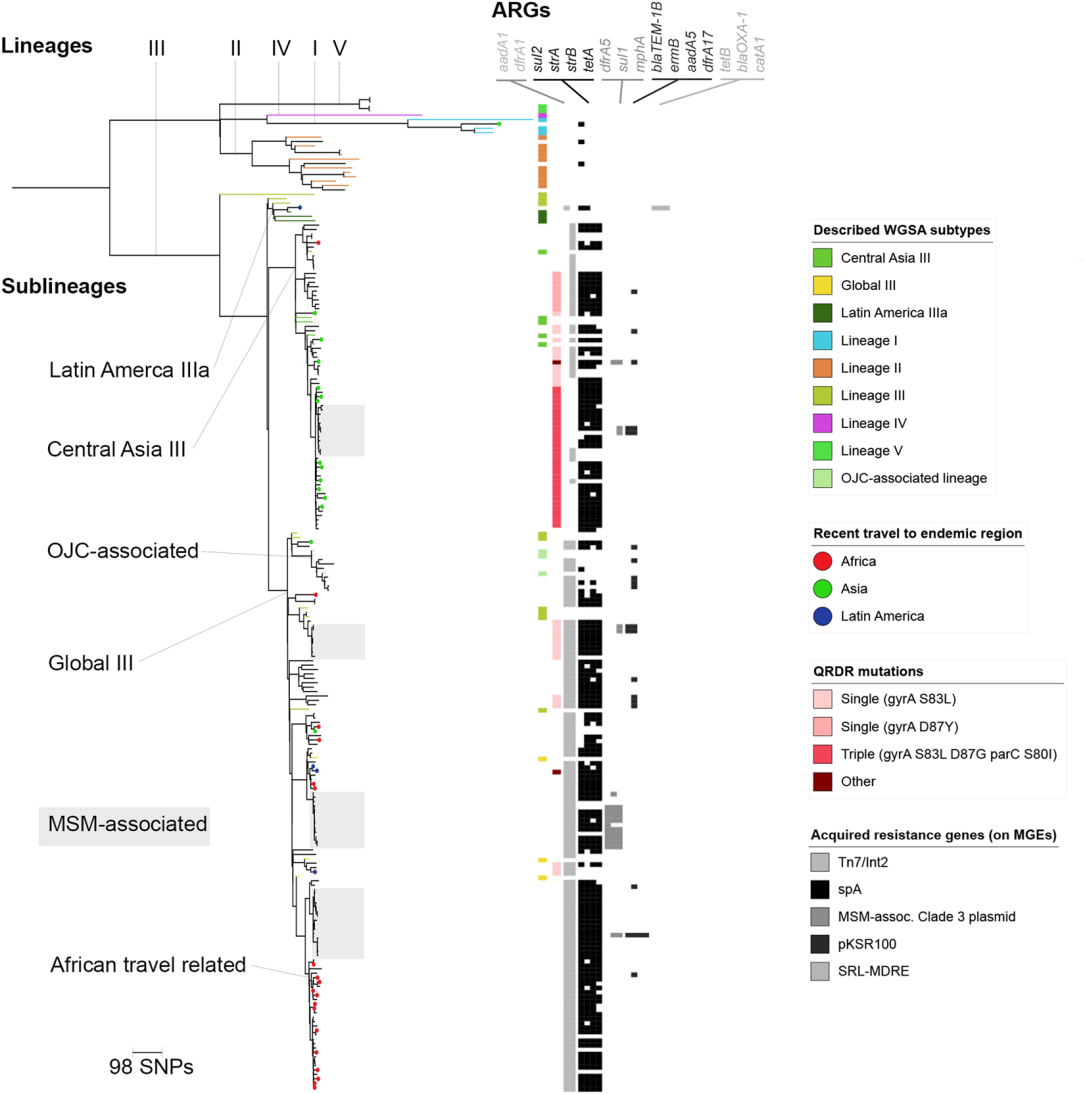
*Shigella sonnei* UK cross section in context. The midpoint-rooted maximum likelihood phylogenetic tree shows isolates from this study in the context of five global lineages of *S*. *sonnei*. Lineage III has further relevant sublineages indicated at the internal nodes and MSM-associated sublineages are highlighted in grey. Isolates from patients recently returned from endemic regions are indicated by circles overlying the tree tips which are coloured according to the inlaid key. Reference isolates from previously described WGSA subtypes (lineages/sublineages) are indicated in the leftmost adjacent track, and tree branches for references isolates are also coloured according to the inlaid key. AMR features are shown in subsequent tracks. The second leftmost track shows the presence of QRDR mutations and two isolates containing ‘Other’ quinolone resistance determinants (including an isolate with a single QRDR mutation and a *qepA* gene, and an isolate containing a *qnrB19* gene). Subsequent gray scale tracks indicate the presence of antimicrobial resistance genes (ARGs, labelled above) associated with known *Shigella* MGEs. Specifically (from left to right), the Tn7/Int2, SpA, MSM-associated sublineage 3 plasmid, pKSR100, SRL-MDRE (Table 1).

For both species, the whole genome phylogenies revealed a dominant subtype. Most *S*. *flexneri* 2a isolates (176 of 179) belonged to PG3 (Fig. 2A) and most (181 of 187) of the *S*. *sonnei* isolates belonged to Lineage III (Fig. 3). For *S*. *flexneri* 2a, this likely represents the strong association of the 2a serotype with the PG3 WGSA subtype. The association was not exclusive though as one PG2 and two PG1 isolates were among the *S*. *flexneri* 2a samples, reaffirming that serotype switching between PGs occurs^8^. For *S*. *sonnei*, the dominance of Lineage III reflects a greater burden of disease caused by this lineage, as previously shown^6^. Despite the Lineage III dominance however, further diversity of *S*. *sonnei* was detected in the UK samples. Specifically, we observed two Lineage V isolates from domestically-acquired cases (despite Lineage V only having been previously reported in Latin America or Africa)^7^, one Lineage I isolate from a patient recently-returned from Asia and three Lineage II isolates from patients without a history of recent travel (Fig. 3). In fact, the only lineage of *S*. *sonnei* not detected, Lineage IV, has yet to be seen outside of its original description^6^.

In addition to this diversity at the highest level of nomenclature (i.e. lineage/PG), analysis at the sublineage level enabled further examination of the role of travel in the epidemiology of shigellosis in the UK. Travel-associated isolates were often phylogenetically proximate to reference isolates from that same region, differing by as few as 3 SNPs (Figs 2B and 3). For *S*. *flexneri* PG3, there was a sublineage related to travel from Asia, containing Asian reference isolates as well as most (11 of the 17) of the patients recently returned from Asia (specific locations were India, Pakistan, Sri Lanka and Afghanistan). To reflect these origins, and mirror *S*. *sonnei* nomenclature, this sublineage was designated *S*. *flexneri* 2a Central Asia (Fig. 2B). The cognate *S*. *sonnei* Central Asia III sublineage (recently described as disseminating from Asia^14^) was similarly detectable (Fig. 3). Specifically, 12 of the 15 isolates of *S*. *sonnei* from patients who had recently travelled to Asia (China and India) belonged to this sublineage. In addition to these Asian-travel related sublineages, there was a similar *S*. *sonnei* sublineage related to travel from Africa (see African travel related, Fig. 3). Specifically, the majority (13 of 19) of isolates from patients who had recently travelled to Africa belonged to a sublineage within the Global III sublineage (Fig. 3). Twelve of these 13 patients had recently returned from Egypt, and one from Nigeria, so it is likely that this sublineage represents the repeated importation of a sublineage that is nationally- or regionally-dominant in Northern Africa. In addition to these Asian- and African-related travel lineages, an *S*. *sonnei* isolate belonging to the Latin America IIIa sublineage was detected in a patient who had recently returned from a Latin American country where this lineage is highly prevalent^7^.

Sublineages transmitting domestically in patient communities were also detectable among the isolates. A separate analysis of this data incorporating patient age and gender identified the six sublineages associated with transmission in MSM (MSM-associated, Figs 2 and 3)^23^. For *S*. *flexneri* 2a there was one large (47 isolates), low-diversity (maximum pair-wise distance 20 SNPs) MSM-associated sublineage, and one smaller (7 isolates), higher-diversity (maximum pair-wise distance 148 SNPs) MSM-associated sublineage (Fig. 2). Among the *S*. *sonnei* isolates, there were four MSM-associated sublineages with maximum pairwise distances of between 15 and 30 SNPs (Fig. 3). One MSM-associated sublineage of *S*. *sonnei* was nested within the Central Asia III sublineage, and three other MSM-associated *S*. *sonnei* sublineages belonged to the Global III sublineage (Fig. 3), which is thought to have disseminated globally after the acquisition of the Tn7/Int2 AMR determinant^6^. In addition to the MSM-associated sublineages, isolates belonging to the Orthodox Jewish community-associated sublineage (also within Global III sublineage) were detected from years spanning 2008–2014 (Fig. 3). This is consistent with a previous study that demonstrates that this Orthodox Jewish community-associated sublineage is circulating between Israel and the UK, where it causes domestically-transmitted outbreaks^10,30^.

### Shared AMR determinants among Shigella species

There were various horizontally-transmissable AMR genes among the isolates, often carried on known MGEs (Table 1). These Antimicrobial Resistance Genes (ARGs) encoded resistance against virtually all known antimicrobial classes. However, genes encoding extended spectrum beta lactamases and plasmid-encoded quinolone resistance genes were rare (10 and 3 of 366 isolates respectively, Supplementary Data). There was a chromosomally-encoded MGE encoding multiple-drug resistance in both *S*. *sonnei* and *S*. *flexneri* (i.e. the Tn7/Int2 determinant and the SRL-MDRE respectively, Table 1, Figs 2 and 3). Notably, the SRL-MDRE was also present in the only *S*. *sonnei* isolate belonging to the Latin America IIIa sublineage (Fig. 3), congruous with the known AMR profile of this sublineage in its endemic region^7^. This isolate also contained the small resistance plasmid pABC-3, typical of Latin America IIIa *S*. *sonnei*^7,31^, and also found in various MSM-associated *Shigella* sublineages (specifically the intercontinentally-distributed MSM-associated *S*. *flexneri* 3a^13^ and the larger sublineage of MSM-associated *S*. *flexneri* 2a (Fig. 2)). Other closely-related small resistance plasmids, specifically the *S*. *sonnei* SpA plasmid, reported in the first sequenced genome^32^, and the resistance plasmid pSFxv_3, originally described in the Chinese epidemic *S*. *flexneri Xv*^33^, were also near-ubiquitous among the isolates (Figs 2 and 3, Table 1). Large resistance plasmids encoding azithromycin, and other, antimicrobial resistances were also found in isolates from MSM-associated sublineages (described more fully elsewhere)^23^.

**Table 1:** Antimicrobial resistance determinants of *Shigella* and species distributions.

Type	Name	Resistance genes	Antimicrobial classes	Species reports
Chromosomal Islands	SRL-MDRE	*tetB* *bla*_OXA-1_ *catA1*	Tetracyclines Ampicillin Chloramphenicol	*S*. *dysenteriae*^15^ *S*. *flexneri*^8,17^ *S*. *sonnei*^7^
	Tn7/Int2	*aadA1* *dfrA1*	Aminoglycosides Trimethoprim	*S*. *flexneri* 2a (this study) *S*. *flexneri Xv*^10,33^ *S*. *sonnei*^6,7^
Small resistance plasmids	pSS046_spA	*sul2* *strA*/*strB* *tetA*	Sulphonamides Streptomycin Tetracyclines	*S*. *sonnei*^6,32^
	pABC-3	*dfrA14* *sul2* *strB* ^^^	Trimethoprim Sulphonamides —	*S*. *sonnei*^7,31^ *S*. *flexneri* 3a^13^ *S*. *flexneri* 2a (this study)
	pSFxv_3	*strA*/*strB* *sul2*	Streptomycin Sulphonamides	*S*. *flexneri Xv*^12^ *S*. *flexneri* 2a (this study)
Large azithromycin resistance plasmids	pKSR100	*mphA* *ermB* *bla*_TEM-1B_ *aadA5* *dfrA17* *sul1*	Macrolides Macrolides Ampicillin Aminoglycosides Trimethoprim Sulphonamides	MSM-associated: *S*. *flexneri* 3a^23^ *S*. *flexneri* 2a^23^ *S*. *sonnei*^23^
	Minor MSMA sublineage plasmid	*mphA* *tetA* *bla*_TEM-1A_	Macrolides Tetracyclines Ampicillin	*S*. *flexneri* 2a^23^
	MSMA sublineage 3 plasmid	*mphA* *dfrA1* *sul1*	Macrolides Trimpethoprim Sulphonamides	*S*. *sonnei*^23^

^^^The *strA* gene has been interrupted by the introduction of *dfra14* on this plasmid meaning no Streptomycin resistance would be conferred.

Owing to its importance for treatment, ciprofloxacin resistance was also examined among the isolates. Remarkably, for both *Shigella* species, triple QRDR mutations conferring resistance to ciprofloxacin were specifically-associated with the Central Asia sublineages (*S*. *flexneri* 2a Central Asia in Fig. 2, Central Asia III in Fig. 3). In addition to these triple-mutant sublineages that evolved in Asia and spread to the UK, isolates and sublineages with single QRDR mutations such as gyrA S83L were present in other parts of the phylogeny (Figs 2 and 3), including in one of the domestically-transmitting MSM-associated *S*. *sonnei* sublineages which contained a gyrA D87Y mutation (Fig. 3). These single mutations have previously shown to be step-wise toward the evolution of fully resistant mutants^14^, and might indicate the potential for ciprofloxacin resistance to evolve in sublineages transmitting locally in the UK.

## Discussion

This study reveals important information about the epidemiology of shigellosis in a high-income nation where shigellosis is frequently related to travel. Indeed, this study confirms that importation of *Shigella* sublineages through travel is a driver of shigellosis in the UK. The most striking examples of this are the repeated importation of sublineages from both Africa and Asia for *S*. *sonnei*, and a similar Asian-travel related sublineage of *S*. *flexneri* 2a. These sublineages likely represent epidemiologically prevailing sublineages circulating in endemic regions that disseminate through travel, as previously shown for the Central Asia III sublineage of *S*. *sonnei*^14^ and for African and Asian-travel related sublineages of *S*. *flexneri* 3a^13^. The importation of sublineages is concerning, particularly when they are resistant to key antimicrobial classes such as the ciprofloxacin-resistant Central Asia sublineages. As our study focused on domestically-acquired shigellosis, we were uniquely able to follow the fate of some of these imported sublineages. Isolates from domestically-acquired cases of shigellosis were present in the travel-related sublineages and likely represent onward transmission from travel-related cases (or alternatively could result from misreporting of travel histories). In our study, domestically-acquired cases of travel-related sublineages often appeared to be dead-end transmissions (i.e. did not form larger clusters of isolates from domestically-acquired infections). This is consistent with recent comprehensive WGSA surveillance of *S*. *sonnei* in the UK where travel-related cases were typically in genetic clusters of only small numbers of isolates^20^. In this study however, we did observe one antimicrobial resistant sublineage that was imported and then went on to contribute to a domestically-transmitting epidemic. Specifically, an MSM-associated sublineage of *S*. *sonnei* was nested within the Central Asia III sublineage, indicating that a newly imported AMR strain can cause domestically-propagated epidemics should they enter an appropriate transmission network)^13,22,23^.

Unfortunately, biases in our subsample (proportional sampling at different depths for travel and non-travel related isolates, as well as age-restrictions for *S*. *sonnei*) make it inappropriate for us to draw quantitative inferences on the relative contribution of this importation to the shigellosis burden in the UK. However, recent results from routine whole genome sequencing surveillance of *S*. *sonnei* in the UK for the period August 2015–January 2016 show that approximately 60% of genetic clusters are likely related to travel^20^.

In addition to directly causing domestically-transmitting epidemics, imported strains can bring in new AMR determinants that might be transferred into locally-circulating sublineages. Encouragingly, there was little evidence for the mobility of mutated QRDRs from imported lineages through chromosomal recombination. However, this was not the case for ARGs carried on MGEs where there was evidence for the exchange of AMR determinants within the UK. For example, the small resistance plasmid carried in the imported Latin America IIIa *S*. *sonnei* (pABC-3) was also found in the domestically-transmitting MSM-associated sublineages of *S*. *flexneri* 2a (Fig. 2) and *S*. *flexneri* 3a^13^ (Table 1). This indicates the potential for imported AMR determinants to horizontally transmit to locally-circulating sublineages and contribute to epidemic emergences. The imported Latin American IIIa *S*. *sonnei* isolate also carried the SRL-MDRE, which is rare in *S*. *sonnei* outside of Latin America, but could mobilise to other pathogens capable of genetic exchange with *S*. *sonnei*. The genetic exchange of AMR determinants between imported and locally-circulating strains means that the public health impact of the importation of AMR pathogens may not be the disease caused by that specific pathogen, but rather the mobilisation of the AMR determinants into other already locally-adapted pathogens and/or patient microbiota, which would then act as a reservoir for AMR emergence. In this way, imported AMR pathogens enrich the UK AMR pool, and may only be noticed only when antimicrobial usage is sufficient to drive their spread to reportable pathogens. Indeed, the ability for horizontal transfer of an AMR plasmid to drive new population-level epidemics is shown in our allied study focused on the MSM-associated sublineages^23^.

The mobility of AMR determinants among *Shigella* sp. is more broadly evidenced by the detection of the same AMR determinants among the diverse global data available. Analysis of previous reports and data from this study show that different *Shigella* species across disparate geographical locations share common AMR MGEs (Table 1). That this mere handful of AMR determinants dominate the genetic landscape of *Shigella* is remarkable considering the breadth of what shigellae (globally ubiquitous, genetically-diverse pathogens capable of lateral gene transfer) would have the opportunity to acquire in the global enteric milieu. The presence of epidemiologically-dominant sublineages with stable AMR determinant associations is commonly interpreted to result from epidemiological success of the sublineage consequent to the conferred AMR phenotype(s). However, in addition to that effect, the small number of recurrent AMR determinants among enormously diverse shigellae might suggest that the success of such AMR determinant-sublineage combinations may also be attributable to some measure of fitness-matching of the AMR determinant for that sublineage/species. It is picking apart these finer details of what limits and drives AMR flow among pathogenic organisms such as *Shigella* that will allow us to understand and perhaps eventually predict new emergences to prevent their occurrence.

## Electronic supplementary material

Dataset 1
